# Intersegment Contacts of Potentially Damaging Variants of Cardiac Sodium Channel

**DOI:** 10.3389/fphar.2021.756415

**Published:** 2021-11-04

**Authors:** Vyacheslav S. Korkosh, Anastasia K. Zaytseva, Anna A. Kostareva, Boris S. Zhorov

**Affiliations:** ^1^ Almazov National Medical Research Centre, St. Petersburg, Russia; ^2^ Sechenov Institute of Evolutionary Physiology and Biochemistry, Russian Academy of Sciences, St. Petersburg, Russia; ^3^ Department of Womenʼs and Childrenʼs Health, Karolinska Institute, Solna, Sweden; ^4^ Department of Biochemistry and Biomedical Sciences, McMaster University, Hamilton, ON, Canada

**Keywords:** Brugada syndrome, cardiac arrhythmias, cryo-EM structure, homology modeling, intersegment contacts, long QT syndrome, Monte Carlo minimizations

## Abstract

Over 1,500 missense variants of sodium channel hNav1.5, which are reported in the ClinVar database, are associated with cardiac diseases. For most of the variants, the clinical significance is uncertain (VUS), not provided (NP), or has conflicting interpretations of pathogenicity (CIP). Reclassifying these variants as pathogenic/likely pathogenic (P/LP) variants is important for diagnosing genotyped patients. In our earlier work, several bioinformatics tools and paralogue annotation method consensually predicted that 74 VUS/NP/CIP variants of 54 wild type residues (set w54) are potentially damaging variants (PDVs). Atomic mechanisms underlying dysfunction of the PDVs are unknown. Here we employed a recent cryo-EM structure of the hNav1.5 channel with likely inactivated pore domain (PD) and activated voltage-sensing domains (VSDs), and *ad hoc* models of the closed and open PD and resting VSDs to explore intersegment contacts of w54 residues. We found that 44 residues from set w54 contact 84 residues with 118 disease missense variants. These include 104 VUS/NP/CIP variants, most of which are associated with the loss-of-function Brugada syndrome (BrS1) or gain-of-function long QT syndrome (LQT3). Matrix representation of the PDVs and their contact variants facilitated recognition of coupled mutations associated with the same disease. In particular, BrS1-associated coupled mutations, which disturb the P-loops region with the selectivity filter slow inactivation gate, would cause the channel dysfunction. Other likely causes of the channel dysfunction include coupled BrS1-associated variants within VSDs that would destabilize their activated states and coupled LQT3-associated variants, which would stabilize the open PD or activated VSDs. Our study proposes mechanisms of channel dysfunction for scores of BrS1- and LQT3-associated variants, confirms status for 82% of PDVs, and suggests damaging status for their contact variants, which are currently categorized as VUS/NP/CIP variants.

## Introduction

Sodium channels Nav1.5 are responsible for initiation and propagation of the action potential in cardiomyocytes. The pore-forming α-subunit of the Nav1.5 channel, which is encoded by gene SCN5A, folds from a single polypeptide chain of four homologous repeat domains (I-IV). Each repeat has six transmembrane (TM) helices (S1-S6) connected by intra- and extracellular loops, including a large extracellular membrane reentering P-loop. The latter contains membrane-descending helix P1 and membrane-ascending helix P2. In each repeat, helices S1 - S4 form a voltage-sensing domain (VSD), while helices S5, S6 and the P-loop contribute a quarter to the pore domain (PD). The selectivity-filter ring DEKA (Asp, Glu, Lys and Ala) divides the ion permeation pathway into two parts: extracellularly exposed outer pore and the inner pore, which in the open channel is exposed to the cytoplasm. Sodium channels adopt various conformations, which in electrophysiological studies are categorized as resting, open, fast inactivated and slow inactivated states.

Sodium channels allow permeation of inward depolarizing current (I_Na_) during phase 0 of the action potential (Amin et al., 2010; Veerman et al., 2015). Nav1.5 dysfunctions cause various hereditary and acquired arrhythmogenic syndromes. Many genetic gain-of-function mutations increase I_Na_ or delay cardiac repolarization, thus prolonging the action potential (Savio-Galimberti et al., 2018). Such mutations are associated with the long QT syndrome (LQT3), a congenital cardiac disorder, which is characterized by prolonged QT interval and higher risk of life-threatening arrhythmia. Clinical manifestations of LQT3 vary from life-long asymptomatic disease course to syncope and cardiac arrest due to torsades de pointes. Mutations, which diminish I_Na_, may decrease cardiac excitability and reduce velocity of electrical conduction, causing various clinical phenotypes such as Brugada syndrome type 1 (BrS1), sick sinus syndrome, sudden infant death syndrome, dilated cardiomyopathy, atrial fibrillation, and arrhythmogenic cardiomyopathy. BrS1 is diagnosed *via* typical ECG-phenomenon with ST-segment elevation in ventricular leads without any structural alterations of the heart (Coppola et al., 2019; Skinner et al., 2019).

Thousands genetic variants of hNav1.5 and other hNav1.x channels are described in public databases, including ClinVar (Landrum et al., 2016), Swiss-Var (Mottaz et al., 2010) and Humsavar (http://www.uniprot.org/docs/humsavar). In April 2021, the ClinVar database reported 1,513 missense variants of SCN5A. Among these, only 17% are classified as benign/likely benign or pathogenic/likely pathogenic (P/LP) variants according to the American College of Medical Genetics and Genomics and the Association for Molecular Pathology (ACMG/AMP) guideline (Richards et al., 2015). Data on pathogenicity is not provided (NP) for 200 variants, conflicting interpretations of pathogenicity (CIP) are reported for 139 variants, and 911 variants are of uncertain clinical significance (VUS).

The ACMG/AMP guideline recommends using computational predictions as a line of evidence for variant interpretation (Richards et al., 2015). Bioinformatics analysis of variants associated with BrS1 suggests that some mutations, which are currently classified as damaging, should be considered benign due to lack of experimental data on channel dysfunction (Kaltman et al., 2018). Incorrect classification of variants is an important problem in medical genetics. Indeed, in many cases a familial history or the pedigree data are lacking. Low frequency of disease-associated genetic variants in population further complicates correct data interpretation and delays diagnostics.

Earlier we employed several bioinformatics tools and the paralogue annotation method to reclassify 74 ClinVar-reported VUS/NP/CIP variants of 54 WT residues (set w54) as potentially damaging variants (PDVs) of hNav1.5 (Tarnovskaya et al., 2020). Here we used a recent cryo-EM structure of the hNav1.5 channel with likely inactivated PD and activated VSDs (Li et al., 2021), and *ad-hoc* models of the open and closed PD and resting VSDs to show that 44 residues from set w54 are involved in 84 intersegment contacts with residues for which 118 missense variants are reported in ClinVar, mostly as VUS/NP/CIP variants. Matrix representation of the PDVs and their contact variants facilitated recognition of coupled variants where substitution of either contact partner is associated with the same disease. We suggest that BrS1-associated variants in P-loops and VSDs would cause loss of channel function, respectively, by disturbing the selectivity-filter slow inactivation gate or destabilizing activated state of VSDs. LQT3-associated variants would cause the channel gain-of-function by increasing population of channels with the open pore. Our analysis confirms the status for most of earlier proposed PDVs, suggests that many of the 118 contact variants of the PDVs may be reclassified as P/LP variants, and proposes atomic mechanisms of loss-of-functions and gain-of function for scores of BrS1-associated and LQT3-associated mutations.

## Methods

### Energy Calculations and Optimization

Our methodology of molecular modeling with the ZMM program is described elsewhere, e.g., (Bruhova and Zhorov, 2010; Korkosh et al., 2014; Tikhonov and Zhorov, 2017). Briefly, we use the AMBER force field (Weiner et al., 1986) and calculate nonbonded interactions with the distance cutoff of 9 Å and a shifting function (Brooks et al., 1985). Electrostatic interactions were calculated with the distance- and environment-dependent dielectric function (Garden and Zhorov, 2010) without the distance cutoff for interactions involving ionized groups. The models were optimized by Monte Carlo energy minimizations (MCM) (Li and Scheraga, 1987) in the space of torsional angles and bond angles of prolines (Zhorov, 1981). MCM trajectories were terminated when 2,000 minimizations did not decrease energy of the apparent global minimum.

### Modeling PD in the Open and Closed States

The hNav1.5 structure with quinidine (PDB ID: 6lqa) captured PD in likely inactivated state and VSDs in the activated states (Li et al., 2021). To model PD in the activated and closed states, we used as templates the open- and closed-pore structures of the NavAb channel (PDB codes 5vb8 and 5vb2, respectively) (Lenaeus et al., 2017). The structures of Nav1.5 and NavAb were 3D aligned by minimizing root mean square deviations of C^α^ atoms in the P1 helices, which are the most 3D-conserved elements of P-loop channels (Tikhonov and Zhorov, 2012), from C^α^ atoms of sequentially matching residues in the Kv1.2/Kv2.1 channel, the first eukaryotic P-loop channel whose crystal structure was obtained at a relatively high resolution below 2.5 Å (PDB ID: 2R9R) (Long et al., 2007). The NavAb and hNav1.5 channels have rather similar folding of the transmembrane (TM) and P1 helices, but different folding of extracellular loops, especially in the PD. Therefore, we imposed distance constraints between C^α^ atoms in the TM and P1 helices of hNav1.5 and matching C^α^ atoms of the template, whereas no distance constraints were imposed to the intra- and extracellular loops. A distance constraint is a flat-bottom parabolic function that imposes the energy penalty with the force constraint of 10 kcal·mol^−1^ Å^−2^ if a C^α^ atom in the model deviates from the template matching atom by more than 2 Å. The 3D alignment was performed by step-wise reducing distances between the C^α^ atoms in the model and template. At each step, the energy was MC-minimized with all degrees of freedom allowed to vary. More methodological details on large-scale transformations of PD can be found elsewhere (Korkosh et al., 2019).

### Modeling VSDs in the Resting States

To model VSDs in the resting states, we employed as a template the crystal structure of the prokaryotic sodium channel NavAb with deactivated VSDs (PDB ID: 6p6x) (Wisedchaisri et al., 2019). Methodology is similar to that used for modeling the channel with the open and closed PD.

### Designations and Visualization of Residues

We designate a residue by one-letter code with superscripted residue number and superscripted prefix indicating the channel segment where the residue is located. Experimental structures and models were visualized with the PyMol Molecular Graphics System, version 0.99rc (Schrödinger, New York, NY).

## Results and Discussions

### Intersegment Contacts in the Cryo-EM Structure

Earlier, we employed several bioinformatics tools and paralogue annotation method to reclassify 74 missense VUS/NP/CIP variants of w54 residues in the hNav1.5 channel as PDVs (Tarnovskaya et al., 2020). Following this publication, eight additional VUS/NP/CIP variants of the w54 residues were reported in ClinVar (version April, 2021). Therefore, currently the PDV set includes 82 variants.

We used a recent cryo-EM structure of the hNav1.5 channel obtained in nanodiscs (Li et al., 2021) to find intersegment contacts of the w54 residues. We instructed PyMol to display 3D neighbor residues within 5 Å from each query residue of set w54. Since the cryo-EM structure lacks hydrogen atoms, the distances were measured between heavy atoms. We have selected those 3D neighbors, which in the amino acid sequence are at least 5 positions away from the query residue and have sidechains facing the query residue sidechain (C^α^ atoms of glycines were considered as sidechains). We further ensured that each 3D neighbor of the query residue has at least one ClinVar-reported missense variant. We found 64 contact residues, which satisfied the above criteria and 85 missense variants of these residues. If two WT residues form a contact, their variants are also considered to contact each other even though they may be farther than 5 Å from each other.

Table 1 shows PDVs and their contact variants in the PD. For each PDV we show location (channel segment), all contact variants, associated syndromes, and their clinical significance. Table 2 shows analogous data for VSDs. Among 85 contact variants, 74 are VUS/NP/CIP variants and clinical significance of 11 variants is P/LP. Four pairs of residues from set w54 are contacting each other (columns “e” in Table 1). Contacts involving channel segments that undergo significant transition upon channel gating are marked “S” in column “f”.

**TABLE 1:** PDVs in the pore domain and their contacts^a^.

PDVs			Contacts				
Variant^b^		Syndrome^c^ ^,^ ^d^	Variant	Syndrome^d^	Significance	^e^	^f^
^IS5^A^242^T		A	^IS6^Q^419^P	BrS1, NP	VUS		S
V		HCM					
^IS5^L^250^V		BrS1	^IS6^V^411^M	LQT3, BrS1, CVP	P		S
^IS5^S^262^G		A, BrS1	^IP1^F^358^S	NS	VUS		
^IS5^V^263^I		Multi	^IVS4^I^1633^V	BrS1	VUS		S
^IS5-P1^G^274^S		BrS1	^IS5-P1^D^356^Y	BrS1	LP		
			N	NP/BrS1	P/LP		
^IS6^A^413^E		LQT3	^IIS6^L^935^P	BrS1	NP		S
S		NS					
T		A, LQT3,BrS1					
^IIS5^G^857^D		A, BrS1	^IIS5-P1^M^881^I	LQT3	VUS	1	
^IIS5-P1^M^881^I		LQT3	^IIS6^F^919^S	NS	LP	1	
			^IIS5^G^857^D	A, BrS1	VUS		
^IIP1^F^892^L		CVP	^IVS6^V^1451^D	BrS1	VUS	2	
I		BrS1	L	NS, A	VUS		
^IIP1^R^893^H		BrS1^1,3,4,8–10^	^IIP2^E^901^K	BrS1	VUS	2	S
C		BrS1					
^IIIS5^V^1324^I		A, BrS1, NP	^IIS3^W^1271^C	BrS1	NP		
^IIIS5^L^1342^F		BrS1	^IIIP1^Y^1409^C	BrS1	NP		S
^IIIP1^L^1410^P		NP	^IIIS5-P2^V^1400^G	CVP	VUS		
^IIIP2^I^1424^F		NP	^IIIS5-P1^F^136^°C	BrS1	NP		
			^IIIS5-P1^V^1400^G	CVP	VUS		
^IIIS6^V^1451^D		BrS1^1,3^	^IIP1^F^892^L	CVP	VUS		
L		A, NS	I	BrS1	NP		
^IIIS6^N^1463^K		NP	^IIS6^F^934^S	BrS1	VUS		
^IIIS6^F^1465^L		LQT3	^IIIS5^V^1337^I	A	VUS		S
			^IIIS5^I^1334^V	LQT3, BrS1, NP	CIP		S
			^IVS6^F^176^°C	BrS1	P		S
			^IVS6^L^1761^F	LQT3	NP		S
			H	LQT3	NP		
^IIIS6^I^1466^T		NP, A	^IIS6^F^934^S	BrS1	VUS		S
V		NP	^IVS6^V^1764^F	BrS1	NP		S
			^IVS6^F^176^°C	BrS1	P		S
^IIIS6^V^1468^A		A, BrS1,NP	^IIIS5^S^1333^Y	LQT3	NP		S
			^IIIS5^V^1337^I	A	VUS		S
^IIIS6^I^1469^F		NP	^IIIS5^I^1334^V	LQT3, BrS1, NP	CIP		S
			^IVS6^L^1761^F	LQT3	NP		S
			H	LQT3	NP		S
			^IVS6^V^1764^F	BrS1	NP		
^IVS5^V^1667^L		NP	^IVS6^I^1758^V	A, BrS1	VUS		
^IVS5-P1^D^1690^N		A, BrS1, NS^14,15,16^	^IP2^R^383^K	BrS1	VUS		
^IVP1^M^1701^I		BrS1, DCM^17^	^IVS5^M^1668^T	A	VUS		
			^IS6^M^390^I	A	VUS		
			V	BrS1, A	VUS		
			^IS6^M^394^I	A	VUS		
			L	A	VUS		
^IVS5^I^1660^V		NP, CVP, BrS1, LQT3^1,3,13,14^	^IVS6^V^1764^F	BrS1	NP	3	S
			^IVS6^L^1761^F	LQT3	NP		S
			H	LQT3	NP		S
			^IIIS4-S5^V^1323^G	BrS1	NP		S
			I	A, BrS1	VUS		S
			^IVS6^M^1766^I	CVP, BrS1	VUS		S
			K	NP, BrS1, SSS	VUS		
			V	BrS1	VUS		
			T	HCM	VUS		
			L	LQT3	P		
			^IIIS6−IVS1^F^1486^L	SIDS	NP		
^IVP1^L^1704^H		BrS1	^IVS5^M^1668^T	A	VUS		
^IVP1^T^1709^M		A, BrS1,CVP,NP^1,3,18^	^IIIP1-P2^K^1419^E	BrS1	NP		
			^IP1-P2^W^374^G	BrS1	NP		
			^IP1-P2^Q^371^R	BrS1	VUS		
			^IS6^I^397^T	BrS1, LQT3, NP	CIP		
^IVS6^M^1766^I		BrS1, CVP	^IS6^N^406^K	LQT3	P, LP	3	S
K		NP, BrS1, SSS	^IS6^N^406^S	BrS1	NP		
V		BrS1	^IVS5^I^1660^V	A, BrS1, CVP, LQT3	CIP		
T		HCM					
L		LQT3					

a *See Energy Calculations and Optimization* Section for definition of contacts.

b All PDVs were listed as VUS in ClinVar-2019. In ClinVar-2021, conflicting interpretations of pathogenicity is reported for three variants (R^894^H, G^1262^D, I^1660^V) and two variants (D^197^G, L^225^V) are classified as likely pathogenic.

c Superscripted numbers refer to clinical and experimental studies of the variants. 1. Huang et al. (2017), 2. Ortiz-Bonnin et al. (2016), 3. Kapplinger et al. (2010), 4. Walsh et al. (2014), 5. Calloe et al. (2013), 6. Liang et al. (2016), 7. Son et al. (2018), 8. Zakliaz’minskaia et al. (2013), 9. Kapplinger et al. (2015), 10. Rudic et al. (2016), 11. Shin et al. (2004), 12. Priganc et al. (2017), 13. Cordeiro et al. (2006), 14. Selga et al. (2015), 15. Núñez et al. (2013), 16. Zeng et al. (2016), 17. Bodian et al. (2017), 18. Li et al. (2018), 19. Brewer et al. (2020).

d Abbreviations: A, Arrhythmia; AF, atrial fibrillation; BrS1, Brugada syndrome; CA, Cardiac Arrest; CIP, Conflicting interpretations of pathogenicity; CVP, Cardiovascular phenotype; DCM, Dilated cardiomyopathy; HCM, Hypertrophic cardiomyopathy; LP, Likely Pathogenic, LQT3, Long QT syndrome Type 3; M, Multiple diseases; NP, not provided; NR, Variants are not reported in ClinVar; NS, Not Specified; P, Pathogenic; PFHB-1, Progressive Familial Heart Block type 1A; PFVF1, Paroxysmal Familial Ventricular Fibrillation 1; SIDS, Sudden Infant Death Syndrome; VUS, Variant of Uncertain Significance.

e Matching numbers show WT residues that are in contact in the cryo-EM structure.

f state-dependent contacts involving residues that move upon activation gating (located C-terminal to gating-hinge in KcsA).

**TABLE 2:** PDVs and their contact variants in the open-PD model^a^.

PDV		Contacts		
Variant	Syndrome	Variant	Syndrome	Significance
^IIIS6^N^1463^K	NP	^IIS6^S^94^°C	BrS1	VUS
		^IIS6^S^941^C	CP	VUS
		N	LQT3	P
^IIIS6^F^1465^L	LQT3	^IIIS5^A^1330^P	LQT3	NP
		T	BrS1, LQT3, More	P/LP
^IIIS6^I^1466^T	NP, A	^IIS6^S^941^C	CP	VUS
V	NP	N	LQT3	P
^IIIS6^V^1468^A	A, BrS1,NP	^IIIS5^A^1330^P	LQT3	NP
		T	BrS1, LQT3, More	P/LP

a Contacts in the cryo-EM structure (Table 1) are not shown.

### Matrices of Contact Variants

**FIGURE 1:**
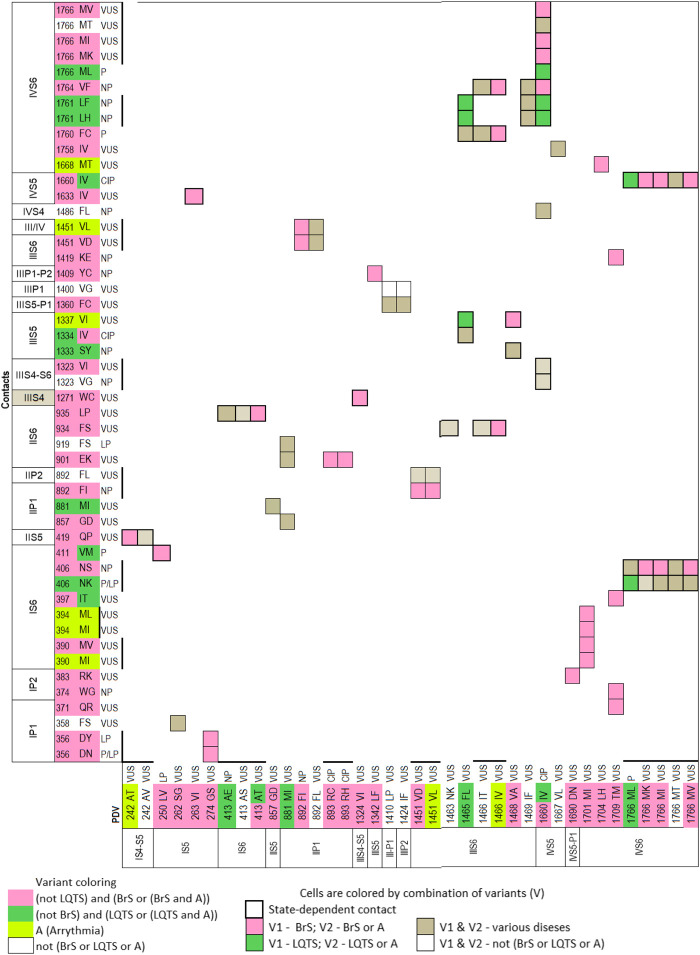
Matrix of contact variants in the PD cryo-EM. Bottom rows (X1-X4) represent w54 residues and their PDVs. X1 and X2 show PD segments and UniProt residue numbers. X3 shows one-letter codes of WT residues and their variants. X4 indicates clinical significance of variants. Variants associated with BrS1, LQT3, non-specified Arrhythmias or other diseases are colored, respectively, pink, green, yellow, or not colored. If besides BrS1 or LQT3, a variant is associated with a non-specified arrhythmia, it is colored pink or green, respectively. If various diseases are reported for a variant, two colors are used. Left columns (Y1-Y4) show analogous features of contact variants of w54 residues. A colored cell in the matrix body links a PDV and its contact variant. If a WT residue has *m* PDVs and *n* contacts variants, a cluster of *m x n* colored cells shows all variant combinations. If PDV and its contact variant have the same color, it is applied to the cell. If a cell links pink and yellow variants, it is colored pink. If a cell links green and yellow variants, it is colored green. If a variant has color A and its contact variants colors A and B, color A is applied to the cell. Otherwise the cell is grey. If a residue is located in cytoplasmic halves of S6 helices, which move upon the channel gating, respective cell is bold-boxed.

To facilitate recognition of disease-related contacts, we schematically represented them in colored matrices. Each matrix represents an individual domain because inter-domain contacts of PDVs are rare. A matrix of contact variants for the inactivated PD (Figure 1) has four bottom rows, which represent PDVs, their location, and clinical significance; four left columns represent analogous characteristics of contact variants of PDVs. Variants associated with BrS1, LQT3 or non-specified arrhythmia are colored pink, green or yellow, respectively. Variants associated with other diseases are not colored. Variants, which are associated with more than one disease, are shown with two colors. Each cell in the matrix body links a PDV and its contact variant.

The matrix highlighted multiple contacts where mutation of either WT residue is associated with the same syndrome. A likely cause is that a disease-related contact contributes to stabilization (or destabilization) of a particular channel state; a weakened or strengthened contact would similarly affect the channel state stability regardless of the mutated contact partner. Structural analysis of all contacts involving PDVs is beyond goals of this study. Below we propose mechanisms of dysfunction for some PDVs and their contact variants.

### Contacts in P-Loops

**FIGURE 2:**
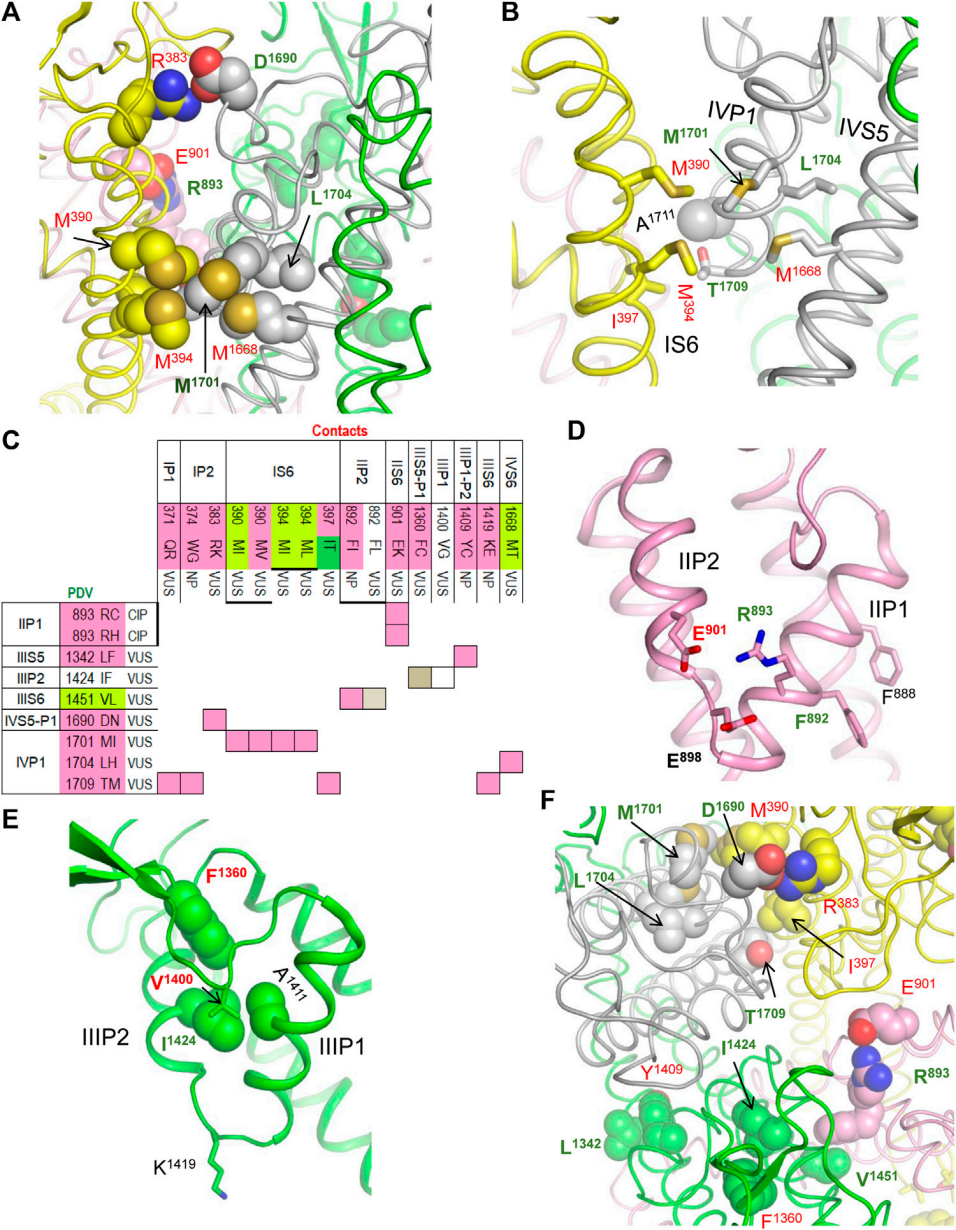
Residue contacts that stabilize P-loops in the cryo-EM structure. Backbones and sidechain carbon atoms in repeats I, II, III and IV are yellow, pink, green and gray, respectively. Residues w54 and their contacts are indicated with green and red labels, respectively. **(A,B)** Intra-membrane views of P-loops in the cryo-EM structure. Residues M^390^, M^394^, M^1668^, M^1701^ and L^1704^ form a tight hydrophobic cluster, which apparently stabilizes mutual disposition of helices IS6, IVS5 and IVP1. **(C)** Matrix of contact variants in P-loops. Majority of coupled variants are associated with BrS1 and arrhythmia, which also may be a loss-of-function disease. **(D)** Intra-membrane view of helices IIP1 and IIP2 whose mutual disposition is stabilized by salt bridge R^893^:E^901^. Side-chain conformation of the selectivity-filter DEKA glutamate E^901^ is stabilized by its salt bridge with R^898^. Helical structure of helix IIP1 is stabilized by π-stacking F^888^ and F^892^. **(E)** Intra-membrane view of helices IIIP1 and IIIP2 whose mutual disposition is stabilized by a cluster of four hydrophobic residues. The selectivity-filter DEKA lysine K^1419^ is shown by sticks. **(F)** Extracellular view of salt bridges R^383^:D^1690^, R^893^:E^901^ and hydrophobic contacts I^397^:T1^709^, L^1342^:Y^1409^ and I^1424^:F^1360^.

In our models with the closed and open PD, which are described in a later section, conformations of P-loops are rather similar to those in the cryo-EM structure. And although P-loops do undergo conformational changes, e.g., upon slow inactivation, here we consider their folding as relatively state-independent. Figure 2 shows P-loops with some contacts, which apparently stabilize their folding and a matrix of respective contacts variants. Five hydrophobic residues (^IS6^M^390^, ^IS6^M^397^, ^IVS5^M^1668^, ^IVP1^M^1701^, and L^1704^) form a tight cluster (Figures 2A,B). In variants M^390^I/V, M^394^I, M^1668^T, and M^1701^I, methionine substitutions with smaller residues (Figure 2C) would relax the cluster and increase flexibility of the smallest involved helix, IVP1. Substitutions ^IVP1^L^1704^H and ^IS6^I^397^T would also increase flexibility of this helix. The C-end of helix IVP1 approaches the DEKA-ring alanine in the SF region (Figure 2B), which harbors the SF gate. The outer-pore of a prokaryotic sodium channel exhibits flexibility during slow inactivation (Chatterjee et al., 2018). In the Nav1.4 channel, the P-loop of domain IV is more flexible than in other domains (Tsushima et al., 1997a; Tsushima et al., 1997b). The above mutations would increase flexibility of helix IVP1, disturb the SF gate, and facilitate the channel transition to the slow-inactivated state. This proposition is supported by the fact that most substitutions in P-loops are associated with the loss-of-function syndromes, especially with BrS1 (Figure 2C).

Several other mutations involving P-loops would also increase their flexibility. For example, in the cryo-EM structure, ^IIP1^F^892^ and ^IIP1^F^888^ form a stacking pair (Figure 2D) that can stabilize the α-helix (Butterfield et al., 2002). Mutation ^IIP1^F^892^L affects the inter-segment contact with ^IIIS6^V^1451^ (Table 1) and destroys the stacking pair with likely destabilization of helix IIP1. A sequential neighbor of F^892^, ^IIP1^R^893^ forms several strong contacts, including a salt bridge with ^IIP2^E^901^, which stabilizes mutual disposition of helices IIP1 and IIP2 (Figure 2D). Substitution of any partner in the salt bridge is associated with BrS1 (Figure 2C) likely due to destabilization of mutual disposition of helices IIP1 and IIP2. Another strong contact of ^IIP1^R^893^ involves the SF glutamate ^IIP1-P2^E^898^, which is proposed to adopt multiple conformations in a cycle of sodium ion permeation (Zhorov, 2021). The cryo-EM structure of Nav1.5 is obtained in complex with quinidine that favors inactivated channels (Li et al., 2021). However, it is unknown whether the SF gate is captured in the open or closed state. A strong electrostatic attraction to ^IIP1^R^893^ may stabilize the E^898^ conformation in the slow-inactivated state.

Several other contacts also stabilize folding of P-loops. An example is a knob-into-hole contact ^IIIP2^I^1424^ : ^IIIP1^A^1411^ that stabilizes the mutual disposition of helices IIIP1 and IIIP2 with the selectivity-filter lysine ^IIIP1-P2^K^1419^ (Figure 2E). Mutation I^1424^F affects contact with A^1411^, as well as contacts with ^IIIS5-P1^F^1360^ and ^IIIS5-P1^V^1400^ whose variants are associated with diseases (Table 1). Another example is a salt bridge ^IVS5-P1^D^1690^ : ^IP2^R^383^ (Figures 2A,F), which is absent in BrS1-associated variants ^IVS5-P1^D^1690^N and R^383^K. In a functional study, variant D^1690^N reduced I_Na_ and shifted the steady-state activation by +7 mV (Zeng et al., 2016).

Thus, majority of mutations affecting the P1 and P2 helices are associated with the loss-of-function BrS1. Apparently these mutations distort the SF gate and increase population of slow-inactivated channels. For comparison, two residues beyond P1 and P2 helices, ^IIS5-P1^M^881^ and ^IIS5^G^857^, form a knob-into-hole contact that would not directly affect the SF region. Both ^IIS5-P1^M^881^I and ^IIS5^G^857^D are PDVs (Table 1). However, unlike the above-described BrS1-associated variants that affect folding of P-helices, M^881^I is associated with the gain-of-function LQT3, while G^857^D is associated with BrS1.

### Contacts in the Channel Models with the Open and Closed PD

**FIGURE 3:**
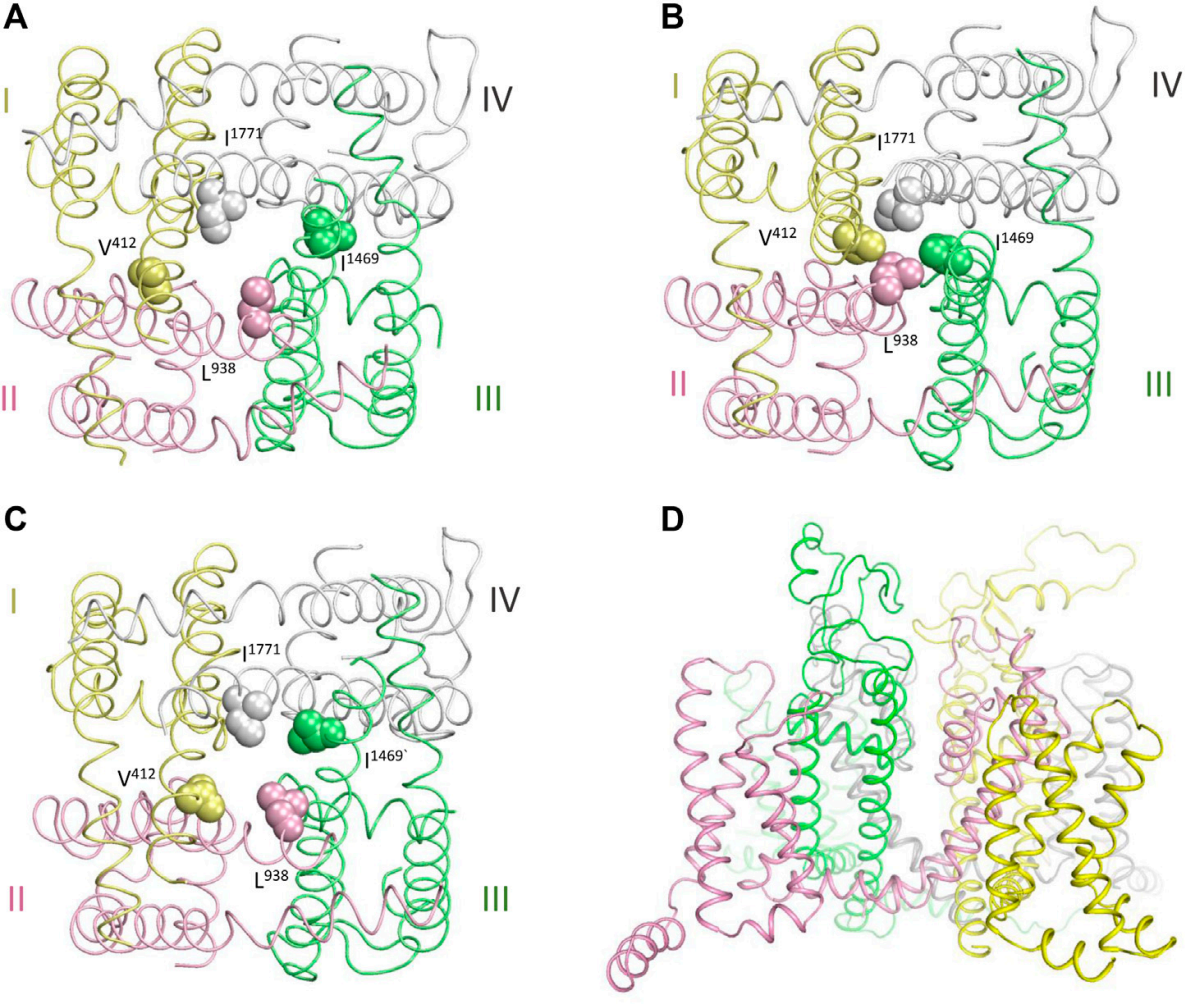
Cryo-EM structure and homology model of the hNav1.5 channel. Repeats I,II, III and IV are yellow, pink, green and gray, respectively. **(A, C)** Cytoplasmic views at the pore domain in the cryo-EM structure **(A)**, and NavAb-based models of the channel states with the closed **(B)** and open **(C)** pore domain. Space-filled residues at the cytoplasmic ends of S6 helices highlight different dimensions of the activated gate. **(D)** Intra-membrane view of the channel model with the open PD.

Figures 3A–C show cytoplasmic views of the PD, respectively, in the cryo-EM structure, closed state, and open state. Space-filled hydrophobic residues at the cytoplasmic ends of S6 helices highlight different dimensions of the activation gate in these states. Figure 3D shows intra-membrane view of the full-fledged channel model with the open PD and activated VSDs. PDVs and respective contact variants, which are specific for the models with the closed PD and open PD, are presented in Tables 2, 3.

**TABLE 3:** PDVs and their contact variants in the closed-PD model^a^.

PDV		Contacts		
Variant	Syndrome	Variant	Syndrome	Signif
^IS6^A^413^E	LQT3	^IVS6^N^1774^D	LQT3	NP
S	NS	S	BrS1	NP
T	A, LQT3,BrS1	Y	NS	CIP
^IIIS6^N^1463^K	NP	^IIS5^S^835^L	BrS1	NP
		A	BrS1	VUS
^IIIS6^F^1465^L	LQT3	^IVS6^I^1768^V	BrS1, LQT3	P
^IIIS6^I^1469^F	NP	^IVS6^I^1768^V	BrS1, LQT3	P
		^IVS6^L^1772^P	BrS1	VUS
		V	LQT3	NP
^IVS6^M^1766^I	CVP, BrS1	^IVS6^G^1661^R	BrS1	NP, CIP
K	NP, BrS1, SSS			
V	BrS1			
T	HCM			
L	LQT3			

a Contacts in the cryo-EM structure (Table 1) are not shown.

**FIGURE 4:**
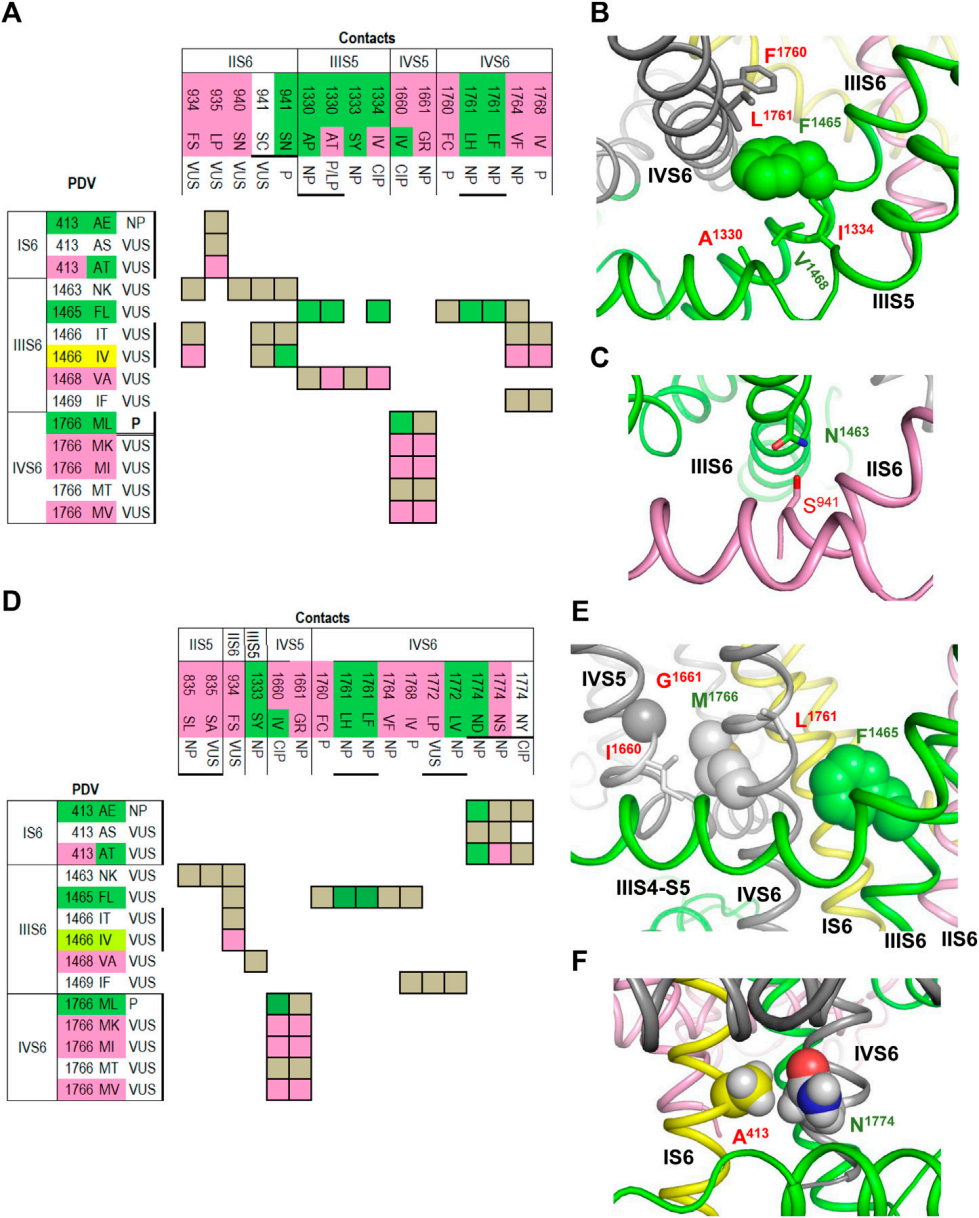
Open and closed conformations of PD. **(A–C)** Open PD model. **(A)** Matrix of contact variants shows only those PDVs and their contacts, which are unique in the open PD model. Most of coupled variants are associated with either BrS1 or LQT3. (**B)** Hydrophobic contacts L^1761^:F^1465^:F^1760^ and A^1330^:V^1468^:I^1334^ stabilize, respectively, mutual disposition of helices IIIS6/IVS6 and IIIS5/IIIS6. **(C)** H-bond S^941^:N^1463^ stabilizes mutual disposition of helices IIS6/IIIS6. **(D–F)** Closed PD model. **(D)** Matrix of contact variants shows only those PDV and their contacts, which are unique in the closed-PD model. **(E)** Mutual disposition of helices IIIS6 and IVS6 in the closed PD is stabilized by hydrophobic contacts F^1465^:L^1761^ with side chain conformations different from those in the open-PD model. In coupled LQT3-associated variants, substitutions F^1465^L or L^1761^F/H would destabilize the closed PD. Mutual disposition of helices IVS5/IVS6 is stabilized by a hydrophobic contact M^1761^:I^1660^ and a knob-into-hole contact M^1761^:G^1661^. **(F)** Mutual disposition of helices IS6/IVS6 is stabilized by a tight hydrophobic contact between A^413^ and methylene group of N^1774^. Replacement of any contact partner with a large residue would destabilize the closed PD, a likely cause of LQT3 associated with these mutations.

Figure 4A shows matrix of contact variants in the cytoplasmic halves of TM helices of the open PD. A hydrophobic cluster involving ^IIIS5^A^1330^, ^IIIS5^I^1334^, ^IIIS6^F^1465^, ^IIIS6^V^1468^ and ^IVS6^L^1761^ (Figure 4B) apparently stabilizes the open PD. Substitutions of A^1330^ with bigger proline or threonine, or substitution of F^1465^ with more flexible leucine are associated with the gain-of-function LQT3 likely because respective variants over-stabilize the open PD. In LQT3-associated variants L^1761^H/F, the open state of PD would be stabilized due to π-stacking of the aromatic substitutions with F^1465^.

Variant ^IVS6^F^1760^C is associated with BrS1. In functional studies, F^1760^C impaired fast inactivation, accelerated recovery from inactivation (Carboni et al., 2005; O'Leary and Chahine, 2015), and enhanced slow inactivation (Carboni et al., 2005). Mutant channel F^1760^A, which is used as a model for drug binding assays, causes a ∼ 20 mV depolarizing shift of steady-state inactivation (Carboni et al., 2005). LQT3-associated mutation A^1330^P increased the window current, whereas mutation A^1330^T, which is associated with LQT3, BrS1 and other diseases, increased the window current, impaired inactivation and accelerated recovery from inactivation (Smits et al., 2005b).

Figure 4C shows the H-bond between ^IIIS6^N^1463^ and ^IIS6^S^941^ that is specific for the open PD. This is an example of inter-repeat H-bonds involving asparagine residues, which are exceptionally conserved in sodium and calcium channels. Earlier such H-bonds were predicted (Tikhonov et al., 2015) and demonstrated (Du et al., 2018) to stabilize the open conformations of these channels. LQT3-associated variant S^941^N would over-stabilize the H-bond and hence the open state of PD, whereas variant S^941^C, which is associated with a non-specific cardiovascular phenotype, may destabilize the H-bond and thus the channel open state. Variant S^941^N was found in an infant died from the sudden infant death syndrome, the disease associated with both Nav1.5 gain-of-function and loss-of-function (Amin et al., 2010). The mutant channel S^941^N expressed in *Xenopus* oocytes demonstrated the increased late current (Schwartz et al., 2000).

Contacts between cytoplasmic halves of TM helices in the closed PD are summarized in matrix shown in Figure 4D. Some open-PD contacts, e.g., ^IVS6^L^1761^ : ^IIIS6^F^1465^, are also seen in the closed PD, but the residue orientations in the two models are substantially different (cf. Figures 4B, E). A knob-into-hole contact ^IVS6^M^1766^ : ^IVS5^G^1661^, which is seen only in the closed-PD model (Table 3), and hydrophobic contact ^IVS6^M^1766^ : ^IVS5^I^1660^ (Figure 4E) apparently stabilize the closed PD conformation. It would be destabilized in LQT3-associated variants M^1766^L and I^1660^V where large hydrophobic residues are substituted with smaller ones. In functional studies, mutation I^1660^V decreased the current, which was rescued by 48 h incubation at room temperature or by mexiletine; the variant was suggested to affect the channel folding, while mexiletine or room temperature stabilized the folding (Cordeiro et al., 2006). Mutation M^1766^L significantly decreased the sodium channel expression, which was partially rescued by mexiletine; however, this mutation also demonstrated a 10-fold increase of the persistent late sodium current (Valdivia et al., 2002).

A tight contact between ^IS6^A^413^ and the methylene group of ^IVS6^N^1774^ is seen only in the closed-PD model (Table 3 and Figure 4F). Substitution of either contact partner with a larger residue would destabilize the closed conformation, resulting in LQT3 (Figure 4D). Mutant channel N^1774^D expressed in the CHO-K1 cells demonstrated increased peak sodium current density, late current and enhanced activation (Kato et al., 2014). Similar electrophysiological changes, as well as markedly increased duration of the action potential, were found in the cardiac myocytes derived from induced pluripotent stem cells from a boy with LQT3-associated mutation N^1774^D (Hirose et al., 2020). Variant N^1774^S was found in a Chinese family with BrS1 (Du et al., 2005).

In the cryo-EM structure, ^IIIS6^F^1465^ forms hydrophobic contacts with four residues whose three variants are associated with LQT3 (Table 1). In our closed-PD model, ^IVS6^I^1768^ forms a tight contact with F^1465^ whose substitution F^1465^L is also associated with LQT3 (Table 3). Substitutions F^1465^L or I^1768^V would destabilize the hydrophobic contact and thus the closed conformation of PD. LQT3-associated substitution I^1768^V would weaken contacts that stabilize both the inactivated and closed-state conformation of the PD. In functional studies, mutant channel I^1768^V accelerated recovery from inactivation and increased the channel availability (Rivolta et al., 2002). In another study, mutant channel I^1768^V accelerated recovery from inactivation, impaired slow inactivation, but did not affect steady-state activation and inactivation (Groenewegen et al., 2003).

### Contacts in VSDs

**FIGURE 5:**
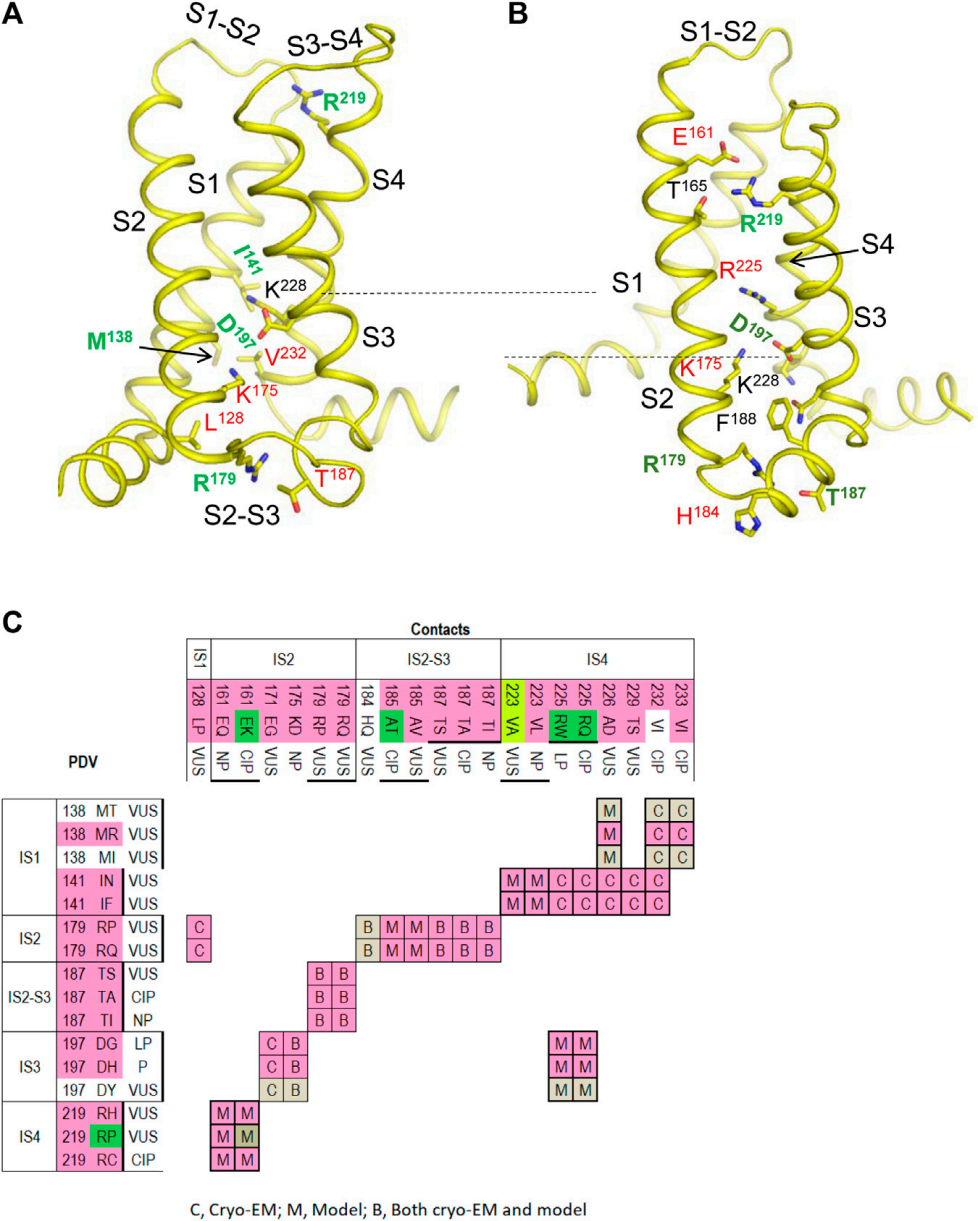
VSD-I in the activated-state structure and resting-state model. **(A,B)** Intra-membrane views at VSD-I in the activated **(A)** and resting **(B)** states. Side chains of some w54 residues and their contacts are indicated with green and red labels, respectively. Dashed lines indicate displacement of K^228^ upon activation of VSD-I. **(C)** Matrix of contacts variants. To indicate contacts, which may switch upon activation of VSD-I, each cell is labeled “C”, “M” or “B” depending on whether the contact is found only in the cryo-EM structure, only in the resting-state model, or both in the cryo-EM structure and the model. Overwhelming majority of PDVs and their contact variants are associated with BrS1. See section *VSD-I* for more details.

**FIGURE 6:**
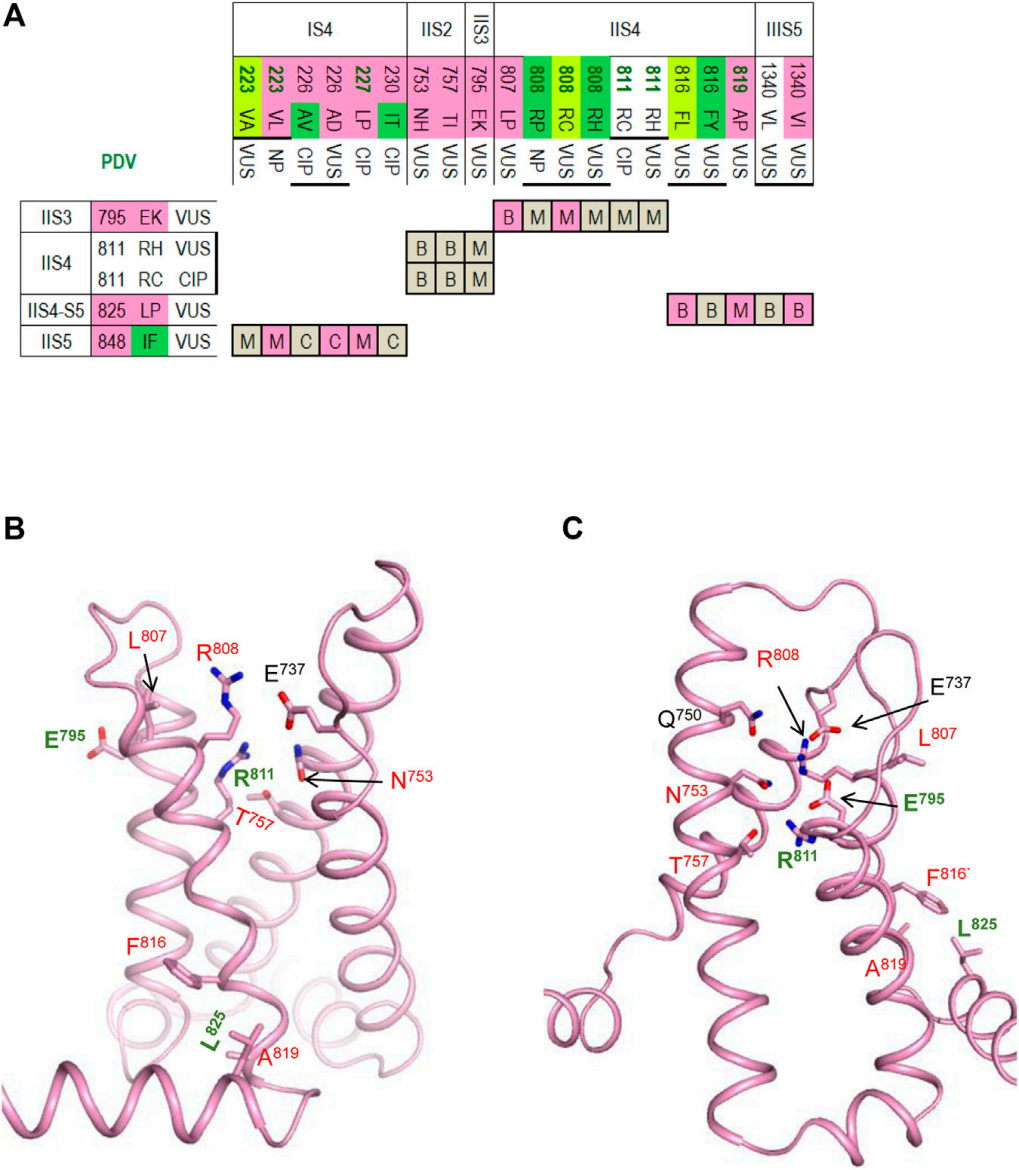
VSD-II in the activated-state structure and resting-state model. **(A)** Matrix of contact variants, in addition to contacts within VSD-II, slows contacts IIS4-5/IIIS5 and IIS5/IS4 **(B,C)** VSD-II in the activated-state cryo-EM structure **(B)** and resting-state model **(C)**. Residues from set w54 and their contacts are indicated with green and red labels, respectively. *VSD-II* Section for more details.

**FIGURE 7:**
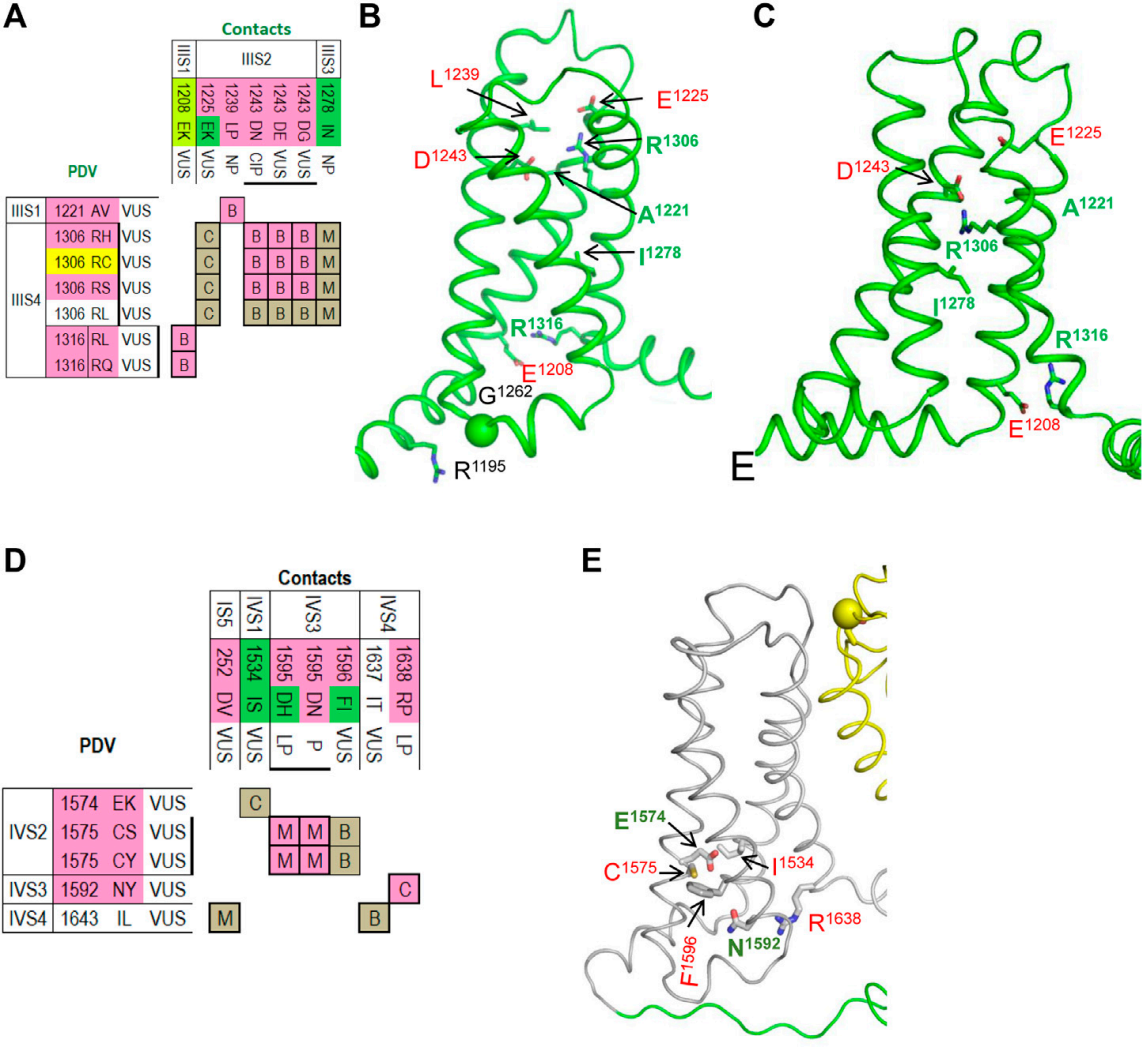
Matrices of contact variants and structures of VSD-III and VSD-VI. **(A)** Matrix of contact variants of VSD-III. **(B,C)** VSD-III in the activated-state cryo-EM structure **(B)** and resting-state model **(C)**. Residues from set w54 and their contacts are indicated with green and red labels, respectively. See section *VSD-III* for more detail. **(D)** Matrix of contact variants of VSD-IV. **(E**) Activated-state structure of VSD-IV. Residues from set w54 and their contacts are indicated with green and red labels, respectively. See section *VSD-IV* for more details.

PDVs and their contacts in the cryo-EM structure with the activated VSDs and models with resting PDVs are shown in Tables 4, 5, respectively. Many contacts switch upon VSD transitions between the activated-state conformations, which are captured in the cryo-EM structure, and resting-state models. We summarized these contacts in four matrices (Figures 5–7) where cells are labeled “C”, “M” or “B” depending on whether respective contacts are seen only in the cryo-EM structure, only in the resting-state model, or both in the cryo-EM structure and the model. It should be noted that ionized residues in VSDs switch contacts during channel gating. Impact of mutations in such contacts on relative stability of different channel states is difficult to predict. On the other hand, respective contacts are promising targets for further studies.

**TABLE 4:** PDVs and their contacts in activated VSDs^a^.

PDVs		Contacts				
Variant^b^	Syndrome^c^	Mutation	Syndrome^d^	Significance	^e^	^f^
^IS1^M^138^I	AF	^IS4^T^229^S	BrS1	VUS		S
T	DCM^1^	^IS4-S5^V^232^I	A, BrS1, CVP	CIP		S
^IS1^I^141^N	BrS1	^IS4^T^229^S	BrS1	VUS		S
F	NS	^IS4^R^225^Q	BrS1, LQT3, More	CIP		S
		W	BrS1, LQT3, More	P/LP		S
		^IS4^A^226^D	BrS1	VUS		S
		V	BrS1, LQT3, More	CIP		
		^IS4^V^232^I	A, BrS1, CVP	CIP		
^IS2^R^179^P	BrS1	^IS1^L^128^P	BrS1	VUS	4	
Q	BrS1	^IS2-S3^T^187^S	BrS1	VUS		
		I	BrS1	NP		
		A	BrS1, CA	CIP		
^IS2-S3^T^187^S	BrS1	^IS2^R^179^P	BrS1	VUS	4	
I	BrS1, NP	Q	BrS1	VUS		
A	BrS1					
^IS3^D^197^G	BrS1	^IS2^K^175^D	BrS1	NP		
H	BrS1	^IS2^E^171^G	BrS1	VUS		
Y	DCM					
^IIS3^E^795^K	BrS1	^IIS4^L^807^P	BrS1, NP	VUS		S
^IIS4-S5^L^825^P	BrS1	^IIS4^F^816^L	A	VUS		S
		Y	LQT3	NP		S
		^IIIS5^V^1340^L	CVP	VUS		
		I	NP, BrS1, A	VUS		
^IIS5^I^848^F	A,BrS1, LQT3^1^	^IS4^A^226^V	BrS1, LQT3, More	CIP		S
		D	BrS1	VUS		S
		^IS4^I^230^T				
^IIIS1^A^1221^V	A, BrS1	^IIIS2^L^1239^P	BrS1	NP		
^IIIS4^R^1306^L	CVP	^IIIS2^D^1243^E	BrS1	VUS		S
H	NP, BrS1	NG	BrS1, More	VUS		S
C	A	^IIIS2^E^1225^K	BrS1	VUS		S
S	BrS1		LQT3, BrS1, A	VUS		S
^IIIS4^R^1316^L	BrS1	^IIIS1^E^1208^K	A, NP	VUS		S
Q	BrS1					
^IVS2^E^1574^K	BrS1^1,3^	^IVS1^I^1534^S	LQT3	VUS		S
^IVS2^C^1575^Y	A, BrS1	^IVS3^F^1596^I	LQT3, BrS1, More	VUS		
S	A					
^IVS3^N^1592^Y	BrS1	^IVS4^R^1638^P	NP	LP		
^IVS4-S5^I^1643^L	NP	^IVS3^I^1637^T	NS	VUS		

^a-e^ footnotes to Table 1.

**TABLE 5:** PDVs and their contacts in resting VSDs^a^.

PDV		Contacts		
Variant	Syndrome	Variant	Syndrome	Signif.
^IS1^I^141^N	BrS1	^IS4^V^223^A	A	LB
F	NP	L	BrS1	NP
^IS2^R^179^P	BrS1	^IS2-S3^H^184^Q	BrS1, CVP	CIP
Q	BrS1	^IS2-S3^A^185^T	BrS1, LQT3, A	CIP
		V	BrS1, A	VUS
^IS4^R^219^C	A, BrS1	^IS2^E^161^Q	BrS1	NP
H	BrS1, DCM	K	BrS1	CIP
P	LQT3, BrS1, More			
^IIS3^E^795^K	BrS1	^IIS4^R^808^P	LQT3	NP
		C	A	VUS
		H	LQT3, A	VUS
^IIS4-S5^L^825^P	BrS1	^IIS4^A^819^P	BrS1	VUS
^IIS5^I^848^F	A, BrS1, LQT3	^IS4^V^223^A	A	LB
		L	BrS1	NP
		^IS4^L^227^P	BrS1	CIP
^IIIS4^R^1306^L	CVP	^IIIS3^I^1278^N	LQT3	NP
H	NP, BrS1			
C	A			
S	BrS1			
^IVS2^C^1575^Y	A, BrS1	^IVS3^D^1595^H	BrS1, LQT3, More	LP
S	A	N	BrS1, More	P
^IVS4-S5^I^1643^L	NP	^IS5^D^252^V	BrS1	VUS
		^IVS3^I^1637^T	NS	VUS

a Contacts in the cryo-EM structure are shown in Table 4.

#### VSD-I

Figure 5 shows VSD-I in the activated (A) and resting (B) states with w54 residues and their contacts. Dashed lines indicate displacement of ^IS4^K^228^ upon transition of the voltage-sensing helix IS4 between the resting and activated states. In the activated VSD-I, R^219^ lacks specific inter-segment contacts, while in the resting VSD-I, R^219^ forms a salt bridge with ^IS2^E^161^ (Figure 5B). BrS1-associated substitutions R^19^C/H/P or E^161^Q/K would eliminate the salt bridge, likely destabilizing the resting VSD-I. Mutation E^161^K, which was found in patients with the sick sinus syndrome, BrS1, or cardiac conduction disease, reduced current density and markedly impaired activation (Smits et al., 2005a).

Variant R^219^H is associated with mixed arrhythmia and dilated cardiomyopathy, likely due to the proton leak current, which was observed in biophysical studies (Gosselin-Badaroudine et al., 2012; Moreau et al., 2018). Mutations R^219^P and E^161^K would destroy the salt ridge and destabilize the resting state, the effect apparently inconsistent with BrS1. However, the same mutations are also associated with LQT3 (Figure 5C).

In the activated VSD-I, ^IS2^R^179^ is H-bonded with ^IS2-S3^T^187^ and forms a hydrophobic contact with ^IS1^L^128^. Variants of these residues are associated with BrS1, indicating that respective mutations destabilize the activated VSD-I. In the resting VSD-I, R^179^ retains the H-bond with T^187^ and interacts with ^IS2-S3^H^184^ and ^IS2-S3^F^188^. Substitutions R^179^P/Q and T^187^S/A/I would eliminate the contacts, thus affecting flexibility of loop IS2-S3. Contact between R^179^ and T^187^ is seen in both activated and resting states of VSD-I, whereas variants of these residues are associated with BrS1, implying that flexibility of loop IS2-S3 is important for activation of VSD-I.

In the activated VSD-I, ^IS3^D^197^ is salt-bridged with ^IS2^K^175^ and ^IS4^K^228^ (Figure 5A). In the resting VSD-I, D^197^ retains the salt bridge with ^IS2^K^175^ and approaches ^IS4^K^228^ and ^IS4^R^225^ (Figure 5B). Substitutions D^197^G/H/T, K^175^N, and R^225^Q/W would eliminate or weaken these contacts in both states. The fact that D^197^G/H/T and K^175^N are associated with BrS1 (Figure 5C) suggests that salt bridge D^197^G : K^175^ contributes to stabilization of the activated VSD-I. Notably, most of variants in VSD-I are associated with BrS1, but four variants are associated with both BrS1 and LQT3 (Figure 5C). The structural cause of the later ambiguity is unclear.

#### VSD-II

The matrix of contact variants in VSD-II (Figure 6A) shows four w54 residues, including ^IIS5^I^848^, which forms state-dependent interdomain contacts with four residues in helix IS4, and ^IIS4-S5^L^825^ that forms interdomain contact with ^IIS5^V^1340^. Most residue substitutions in the interdomain contacts are associated with BrS1, but three variants are also associated with LQT3. Homozygous mutation ^IIIS5^V^1340^L was found in two female patients with atrial standstill, while in three unaffected relatives the mutations was heterozygous. In a functional study, V^1340^L reduced the current density and impaired channel activation (Tan et al., 2018). Another mutation, V^1340^I, did not alter biophysical characteristics at room temperature, but at 40°C it decreased the current density, caused hyperpolarizing shift of steady-state inactivation, and accelerated recovery from the fast inactivation (Samani et al., 2009).

The activated VSD-II is stabilized by a salt bridge ^IIS4^R^808^ : ^IIS1^E^737^, an H-bond ^IIS4^R^811^ : ^IIS2^N^753^, a hydrophobic contact ^IIS3^E^795^ : ^IIS4^L^807^, and hydrophobic contacts involving ^IIS4^F^816^, ^IIS4^A^819^ and ^IIS4-S5^L^825^ (Figure 6B). The latter contacts are also seen in the resting VSD-II, but the downshift of helix IIS4 significantly rearranged contacts involving R^808^ and R^811^ (Figure 6C). Salt bridges R^808^ : E^737^, R^811^ : E^795^ and an H-bond R^808^ : E^795^ apparently stabilize the resting VSD-II. Mutations R^808^P/H would eliminate the salt bridges and destabilize the resting state of VSD-II stronger than its activate state in which R^808^ forms only one salt bridge. This model-based prediction agrees with the fact that variants R^808^ P/H are associated with LQT3 (Figure 6A).

#### VSD-III

The matrix of contact variants in VSD-III shows seven PDVs and seven their contact variants (Figure 7A). Activated VSD-III is stabilized by a salt bridge ^IIIS4^R^1306^ : ^IIIS1^E^1225^, which is seen only in the cryo-EM structure, two salt bridges seen in both the cryo-EM structure and resting-state model (R^1306^ : ^IIIS2^D^1243^ and ^IIIS4^R^1316^ : ^IIIS1^E^1208^) and the hydrophobic contact ^IIIS2^L^1239^ : ^IIIS1^A^1221^ (Figure 7B). Elimination of the salt bridges in these variants is usually associated with BrS1 (Figure 7A). In the cryo-EM structure, ^IIS1^R^1195^ and ^IIS2-S3^G^1262^ are farther than 5 Å from each other (Figure 7B), but PDV G^1262^S (Table 6) may form an H-bond with R^1195^ and decrease flexibility of linker IIS2-S3.

**TABLE 6:** PDVs whose missense contact variants in the cryo-EM structure or models are not listed in ClinVar.

#	PDV		Exposure	Comment
	Variant^a^	Syndrome^b,c^		
1	^IS6^V^396^L	BrS1^1,3,4^	Lipids	Tight contacts with^IS5^F^264^ ^IP1^L^365^ and ^IP1^L^368^
2	^IIS1^L^732^P	BrS1		
3	^IIS1-S2^L^736^P	BrS1	Extracellular	Loop Flexibility
4	^IIS1-S2^H^738^P	BrS1	Extracellular	Loop Flexibility
5	^IIS3^M^794^T	NP	Lipids	
6	^IIS4-S5^G^833^R	LQT-3,NP,A, BrS1^7^	Lipids	
7	^IIS5^V^924^F	NP	Lipids	
8	^IIIS1^R^1195^C	BrS1	Cytoplasm	R^1195^ may reach G^1262^ “
9	^IIIS1^R^1195^H	“	Cytoplasm	
10	^IIIS2-S3^G^1262^S	BrS1, LQT-3, PFHB^1,3,11,12^	Cytoplasm	Loop flexibility
11	^IIIS6^I^1448^L	A, BrS1, CVP^1,3,4^	Lipids	

^a-e^ See footnotes to Table 1.

The resting state of VSD-III is stabilized by a hydrophobic contact of I^1278^ with methylene groups of R^1306^ (Figure 7C). Variant I^1278^V, which was found in a Thai patient with congenital complete heart block, is considered disease-causative because this variant was not found in unaffected members of the patient’s family (Thongnak et al., 2016). Substitution I^1278^N would destabilize the resting state in agreement with the fact that this variant is associated with LQT3 (Figure 7A).

#### VSD-IV

The matrix of contact variants in VSD-IV shows five PDVs and seven their contacts variants (Figure 7D). Several contacts at the cytoplasmic half of VSD-IV stabilize the activated state (Figure 7E); weakening or elimination of these contacts in BrS1-associated PDVs would destabilize the activated VSD-IV. In contrast, LQT3-associated mutation ^IVS1^I^1534^S would stabilize the activated state due to H-bond between ^IVS2^E^1574^ and ^IVS1^I^1534^S. In both activated and resting states of VSD-IV, ^IVS3^F^1596^ forms a hydrophobic contact with ^IVS2^C^1575^ (Figure 7D). Variants C^1575^S/Y are associated with BrS1, whereas variant F^1596^I is associated with both BrS1 and LQT3. In functional studies, F^1596^I did not change peak current, but enhanced steady-state inactivation and accelerated recovery from inactivation (Olesen et al., 2012; Hoshi et al., 2015).

### Domain Distribution of PDVs and their Contact Variants

Table 7 shows domain distribution of PDVs, their contact variants, and clinical significance of the latter variants. In the PD, 27 WT residues with a total of 38 PDVs are engaged in contacts with 45 WT residues, which have a total of 63 missense disease variants. In the four VSDs, 17 WT residues with a total of 33 PDVs contact 39 WT residues with a total of 55 missense disease variants. While majority of PDVs and their contact variants are seen in the cryo-EM structures, models of the open/closed PD and resting VSDs revealed an additional PDV and 34 contact variants. Thus, in the considered channel states, w54 residues with 82 PDVs form contacts with 84 WT residues that have a total of 118 disease variants. Current number of PDVs exceeds the published number (Tarnovskaya et al., 2020) because new variants of w54 residues were recently reported in ClinVar. For majority of the published PDVs clinical significance is still VUS/NP/CIP, but two variants, ^IS5^L^250^V and ^IVS6^M^1766^L, were recently reclassified as P/LP. As many as 104 contact disease variants are categorized as VUS/NP/CIP variants. Further bioinformatics analysis is required to predict potentially damaging effect of these variants, but the fact that they are coupled with earlier predicted PDVs suggests that a large portion of the 104 variants would be reclassified as a P/LP variants.

**TABLE 7:** Domain distribution of PDVs and their contact variants.

PDVs				Contacts		Clinical significance of contact variants			
Domain	State	WT	Variants	WT	Variants	VUS	P/LP	NP	CIP
PD	Inactivated^a^	27	38	37	49	29	7	11	2
	Open^b^			3	5	2	2	1	
	Closed^b^			5	9	2	1	5	1
VSD-I	Activated^a^	5	13	11	15	8	1	2	4
	Resting^c^	1	3	3	6	2		2	2
VSD-II	Activated^a^	4	5	8	10	8			2
	Resting^c^			6	10	6		2	2
VSD-III	Activated^a^	3	7	4	6	4		1	1
	Resting^c^			1	1			1	
VSD-IV^d^	Activated^a^	4	5	4	4	3	1		
	Resting^c^			2	3	1	2		
Total		10	11						
		54	82	84	118	65	14	25	14
Set^e^		**w54**	**PDV**	**c84**	**cv118**				

a Cryo-EM structure.

b WT residues that have contacts variants in the inactivated PD (cryo-EM structure) are not counted.

c WT residues that have contacts variants in the activated VSD (cryo-EM structure) are not counted.

d Location is not shown for WT residues that do not form contacts with disease-associated residues.

e Names of the sets are shown in bold.

Table 8 shows domain distribution of disease variants. Most of these are associated with BrS1, followed by LQT3 and unspecified arrhythmia. A total of 32 variants are associated with other diseases, which are specified in Tables 1–5. In the contact variant matrices respective variants are not colored. It should be noted that most of “other” diseases are associated with the Nav1.5 loss-of-function. The cases when variants associated with “other” loss-of function diseases are coupled with BrS1-associated variants provide additional examples of contacts in which mutation of either partner destabilizes the open channel.

**TABLE 8:** Domain distribution of disease variants.

Location	State	BrS1		LQT3		Arrythmias		Other diseases	
		PDV	Contacts	PDV	Contacts	PDV	Contacts	PDV	Contacts
PD	Inactivated ^a^	21	30	6	10	3	6	10	7
	Open ^b^		2		3				1
	Closed ^b^		6		3				1
VSD1	Activated ^a^	10	14		2			3	2
	Resting ^c^	3	5	1	2		1		
VSD2	Activated ^a^	3	7	1	3		1	2	1
	Resting ^c^		4		2		2		2
VSD3	Activated ^a^	5	5		1	1	1	1	
	Resting ^c^				1				
VSD4	Activated ^a^	4	2		2			1	1
	Resting ^c^		3		1				
Total		46	78	8	30	4	11	17	15

^a-c^ See footnotes to Table 7.

Only eleven of 82 PDVs (13%) are not coupled with other disease variants (Table 6). One PDV (^IS6^V^396^L) is involved in tight intra-PD contacts, while 10 PDVs are exposed to lipids, cytoplasm or extracellular space. Three variants (^IIS1-S2^L^736^P, ^IIS1-S2^H^738^P, and ^IIIS2-S3^G^1262^S) affect flexibility of VSD linkers. The fact that 87% of PDVs are coupled with disease variants supports bioinformatics-based predictions of PDVs (Tarnovskaya et al., 2020) and suggests that many contact variants of PDVs, which are currently annotated as VUS/NP/CIP, may be reclassified as P/LP variants.

### Limitations of the Approach

In the absence of cryo-EM structures of the hNav1.5 channel with the open and closed pore domain and resting VSDs, we modeled these states using as templates crystal structures of prokaryotic sodium channel NavAb (see Methods). Some of the proposed intersegment contacts in these states require future validation in functional studies. Ionizable residues in VSDs are involved in multiple state-dependent electrostatic interactions. Structural interpretation of respective mutations may be ambiguous.

## Concluding Remarks

Only a small fraction of known missense variants of the hNav1.5 channel are classified as P/LP variants. Prediction of damaging effects of VUS/NP/CIP variants would improve diagnostics of increasingly large number of genotyped cardiac patients. Here we employed a recent cryo-EM structure of the Nav1.5 channels and *ad-hoc* homology models of the channel with the open or closed PD and resting VSDs to explore intersegment contacts of 54 WT residues whose VUS/NP/CIP variants are reclassified as PDVs (Tarnovskaya et al., 2020). We found that 44 of the 54 WT residues form intersegment contacts with 84 WT residues for which 118 disease variants are reported. Overwhelming majority of the contact variants is categorized as VUS/NP/CIP. We propose that if two WT residues, which are engaged in an intersegment contact, have VUS/NP/CIP variants, these are P/LP variants. Validation of this proposition requires bioinformatics analysis of a large set of contacts involving disease variants and electrophysiological testing of some contacts (Smith and Goldin, 1997; Bénitah et al., 1999). In many contacts, variants of either residue are associated with the same syndrome likely because they similarly affect stability of a particular channel state. To facilitate recognition of such coupled variants, we presented them in colored matrices. Structural analysis suggested atomic mechanism of the channel dysfunction in scores of coupled variants. These include BrS1-associated mutations in P-loops that would destabilize the selectivity-filter slow inactivation gate, BrS1-associated variants in VSDs that would destabilize their activated states, and LQT3-associated mutations in PD that would stabilize the channel states with the open pore (or destabilize states with the closed pore). Understanding these mechanisms may assist structure-based predictions of pathogenic variants and contribute to general physiology of ion channels.

## Abbreviations

BrS1, brugada syndrome type 1; CIP, conflicting interpretations of pathogenicity; LQT3, long QT syndrome type 3; MC, monte carlo; MCM, MC-minimizations; NP, data on pathogenicity are not provided; P1 and P2, membrane-descending and membrane-ascending pore helices in P-loops; PD, pore domain; P/LP, pathogenic/likely pathogenic; PDV, potentially damaging variant (P/LP and PDV are used, respectively, for database-listed variants and those predicted computationally); SF, selectivity filter; TM, transmembrane; VSD, voltage-sensing domain; VUS, variant of uncertain clinical significance; w54, 54 wild-type residues for which PDVs are predicted; WT, wild type.
